# Temporary Mechanical Circulatory Support in Patients with Cardiogenic Shock: Clinical Characteristics and Outcomes

**DOI:** 10.3390/jcm12041622

**Published:** 2023-02-17

**Authors:** Michael Abiragi, Tahli Singer-Englar, Robert M. Cole, Dominic Emerson, Fardad Esmailian, Dominick Megna, Jaime Moriguchi, Jon A. Kobashigawa, Michelle M. Kittleson

**Affiliations:** 1 Department of Medicine, Cedars-Sinai Medical Center, Los Angeles, CA 90048, USA; 2 Department of Cardiology, Smidt Heart Institute, Cedars-Sinai Medical Center, Los Angeles, CA 90048, USA; 3 Department of Cardiac Surgery, Smidt Heart Institute, Cedars-Sinai Medical Center, Los Angeles, CA 90048, USA

**Keywords:** heart failure, cardiogenic shock, cardiac transplantation, mechanical circulatory support

## Abstract

Patients with cardiogenic shock may require stabilization with temporary mechanical circulatory support (tMCS) to assess candidacy for definitive therapy, including heart transplantation (HTx) or durable MCS, and/or maintain stability while on the HTx waiting list. We describe the clinical characteristics and outcomes of patients with cardiogenic shock who underwent intra-aortic balloon pump (IABP) vs. Impella [Abiomed, Danvers, MA, USA] placement at a high-volume advanced heart failure center. We assessed patients ≥ 18 years who received IABP or Impella support for cardiogenic shock from 1 January 2020 to 31 December 2021. Ninety patients were included, 59 (65.6%) with IABP and 31 (34.4%) with Impella. Impella was used more frequently in less stable patients, as evidenced by higher inotrope scores, greater ventilator support, and worse renal function. While patients on Impella support had higher in-hospital mortality, despite the worse cardiogenic shock in patients for whom clinicians chose Impella support, over 75% were successfully stabilized to recovery or transplantation. Clinicians elect Impella support over IABP for less stable patients, though a high proportion are successfully stabilized. These findings demonstrate the heterogeneity of the cardiogenic shock patient population and may inform future trials to assess the role of different tMCS devices.

## 1. Introduction

Heart transplantation (HTx) remains the definitive therapy for patients with end-stage heart disease. However, some patients with cardiogenic shock require stabilization with temporary mechanical circulatory support (tMCS) to improve end-organ function, assess candidacy for definitive therapy, and/or maintain stability while on the HTx waiting list. Options for stabilization include an intra-aortic balloon pump (IABP) or catheter-based microaxial temporary ventricular assist device (Impella device, Abiomed, Danvers, MA). Randomized trials of IABP vs. Impella support show comparable outcomes [1,2,3], though these trials are limited by small sample size, a narrow focus on cardiogenic shock after acute myocardial infarction, and the use of Impella 2.5 [1] or Impella CP [2,3] devices as opposed to the Impella 5.5 which offers greater circulatory support.

Observational analyses of Impella vs. IABP in cardiogenic shock offer variable conclusions. In cardiogenic shock post-acute myocardial infarction, there was higher mortality in patients receiving Impella support [4], but in heart transplant recipients supported with Impella vs. IABP, post-transplant survival was comparable [5].

The optimal strategy in patients with cardiogenic shock who may be considered candidates for heart transplantation or durable MCS remains unclear. With the increased use of tMCS as a bridge to transplantation following the 2018 revised UNOS allocation policy [6], a better understanding of clinicians’ choices in the management of cardiogenic shock with tMCS is essential. The purpose of this study was to describe the clinical characteristics and outcomes of patients with cardiogenic shock who undergo IABP vs. Impella placement at the discretion of the treating clinicians at a high-volume advanced heart failure center.

## 2. Materials and Methods

The patient population inclusion criteria comprised patients ≥ 18 years of age who were initiated on IABP or Impella support for cardiogenic shock from 1 January 2020 to 31 December 2021, at a large center, identified by procedure charge codes, who were cared for during admission by an advanced heart failure/transplant cardiologist. Patients were excluded if Impella CP was utilized, tMCS was placed for high-risk percutaneous coronary intervention, unplanned deterioration in the cardiac catheterization laboratory, or for left ventricular venting in a patient on extracorporeal membrane oxygenation.

Demographic and clinical information was obtained via chart review of a prospectively maintained database utilizing the hospital-based electronic medical record. Baseline demographic and clinical characteristics were collected at the time of tMCS initiation.

Outcomes assessed include tMCS complications and longer-term outcomes of morbidity as well as mortality. Complications assessed include any bleeding, limb ischemia, vascular complications (pseudoaneurysm, AV fistula, vessel thrombosis/distal embolization, vessel dissection, perforation or rupture, vessel stenosis, cannulation site bleeding, and vascular access site infection), major hemolysis, minor hemolysis, infection adverse event, stage 3 acute kidney injury (AKI), need for renal replacement therapy (RRT) due to stage 3 AKI, need for RRT due to chronic renal dysfunction, type 1 neurologic dysfunction adverse event (acutely symptomatic central nervous system injury), and major device malfunction. All complications were defined by the criteria outlined in the MCS academic research consortium consensus statement [7]. Minor hemolysis is defined as laboratory evidence of hemolysis without clinical signs, symptoms, or pump malfunction, whereas major hemolysis is laboratory evidence of hemolysis plus at least one of these features [7]. Outcomes included duration of support, recovery, transition to durable MCS, heart transplantation, in-hospital mortality, and all-cause mortality.

Clinical characteristics and outcomes were compared between patients supported with IABP and patients supported with Impella. Categorical variables were reported as percentages and compared using Fisher’s exact test. Continuous variables were reported as median (25th–75th percentile) and compared with the Mann–Whitney test. Adverse events were reported as the number of incidences per 100 person-years and compared via Poisson distribution.

Kaplan–Meier survival analysis was performed for freedom from mortality estimates and a Fine–Gray competing risk model was generated to assess for risk of mortality with transplantation and LVAD as a competing event. Competing risks analysis was performed to compare time to the first outcome of death, transplant, or LVAD between groups. Statistical analysis was performed using SAS software, Version 9.4 (SAS Institute Inc., Cary, NC, USA).

## 3. Results

### 3.1. Patient Population

**Figure 1:**
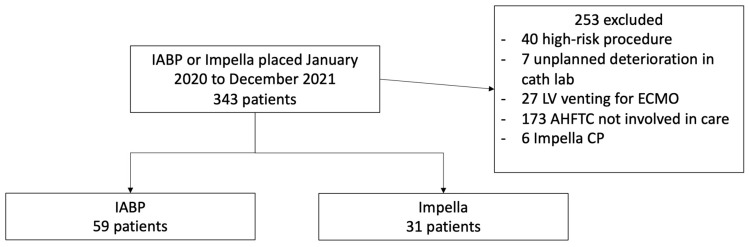
Cohort derivation flow chart. AHFTC, advanced heart failure and transplant cardiologist; IABP, intra-aortic balloon pump; ECMO, extracorporeal membrane oxygenation; LV, left ventricular; PCI, percutaneous coronary intervention.

Between January 2020 and December 2021, 343 patients had an IABP or Impella placed (Figure 1).

Of those, a total of 253 were excluded due to tMCS placement: (a) pre-emptively for high-risk percutaneous coronary intervention, coronary artery bypass grafting (CABG), or VT ablation (*n* = 40); (b) tMCS placement for unplanned deterioration in the cardiac catheterization laboratory, during CABG or OHT (*n* = 7); (c) left ventricular venting for patients receiving extracorporeal membrane oxygenation support (*n* = 27); (d) not being followed by an advanced heart failure-transplant cardiologist (*n* = 173); or (e) utilization of the Impella CP (*n* = 6).

The cohort for subsequent analysis included 90 patients, 59 (65.6%) with IABP and 31 (34.4%) with Impella support. Impella support included 1 (3.2%) Impella 5.0, and 30 (96.8%) Impella 5.5. Table 1 describes the clinical characteristics of patients at the time of tMCS placement.

There were no differences in age or race/ethnicity between groups. Patients with IABP were more likely to be female (23.7% vs. 6.5%, *p* = 0.047). Clinicians elected for Impella support in patients who had higher body mass index (30.2 kg/m^2^ vs. 24.2 kg/m^2^, *p* < 0.001). The underlying etiology of cardiomyopathy was similar between groups, though clinicians chose Impella to support more often for patients in cardiogenic shock post-myocardial infarction (16.1% vs. 1.7%, *p* = 0.03) and less often in patients with shock from acute decompensated heart failure (83.9% vs. 98.3%. *p* = 0.017). There were no significant differences in the proportion of patients with diabetes mellitus or renal replacement therapy.

There were no significant differences in ejection fraction, systolic blood pressure, right atrial pressure, pulmonary artery pressures, pulmonary capillary wedge pressure, or cardiac index. However, patients for whom clinicians chose Impella support had a higher inotrope score (6.9 vs. 6.0, *p* = 0.005), though it is not clear whether this is a clinically relevant difference given the lack of significant differences in dosages of individual inotropic agents. The lack of observed elevation in lactate levels may reflect the fact that these patients were already stabilized with inotropic support prior to tMCS placement. While there was no difference in the use of CPR at the time of tMCS initiation between groups, patients in the Impella group were more likely to be on ventilator support (16.1% vs. 0%, p< 0.001) at the time of tMCS and had higher creatinine levels (1.7 mg/dL vs. 1.2, *p* = 0.004). There was no difference in the INTERMACS profile at the time of tMCS initiation.

### 3.2. Temporary MCS Complications

Table 2 describes MCS complications among patients in both groups.

There was no difference in the event rates of the majority of temporary MCS complications between patients with Impella versus IABP support, including no difference in cannulation site bleeding, minor hemolysis, infections, neurologic events, or major device malfunction.

Patients for whom clinicians chose Impella support experienced lower rates of limb ischemia (expected as an axillary approach was used) but higher rates of major hemolysis and acute kidney injury. Both complications would be expected given the larger cannula size, mechanism of action, and less stable patient population who received Impella support.

### 3.3. Outcomes

Table 3 describes the acceptable clinical outcomes of the two groups of patients.

**Figure 2:**
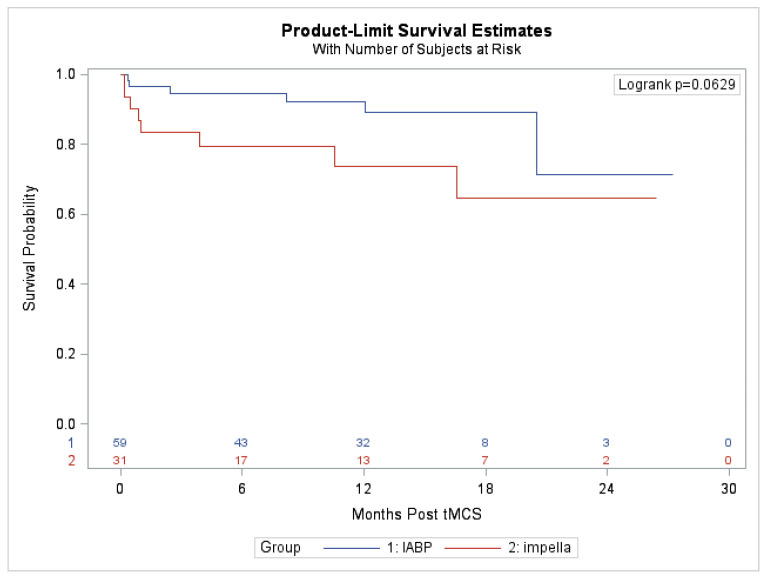
Survival analysis for patients with Impella vs. IABP placed for cardiogenic shock.

Compared with patients supported with IABP, patients with Impella support had a longer median duration of support (15 vs. 7 days, *p* < 0.001). Patients with Impella support also had a higher in-hospital mortality (19.4% vs. 3.4%, *p* = 0.018); however, there was no significant difference in all-cause mortality over the course of follow-up. Figure 2 depicts a survival analysis for patients in the two groups.

**Figure 3:**
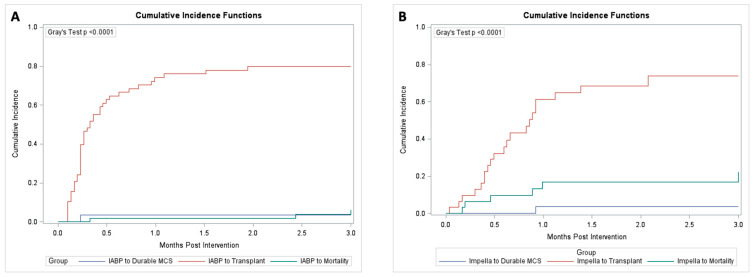
(**A**) Competing event model of outcomes for IABP for mortality, transplant, and durable MCS; (**B**) Competing event model of outcomes for Impella for mortality, transplant, and durable MCS.

There was no significant difference in the proportion of patients that experienced recovery (17% vs. 9.7%; *p* = 0.53) transitioned to durable MCS (3.4% vs. 3.2%; *p* = 1.0; Figure 3), or underwent heart transplantation (67.7% vs. 76.3%, *p* = 0.455).

## 4. Discussion

In patients with cardiogenic shock at a high-volume advanced heart failure center, clinicians were more likely to choose IABP support for patients not on ventilatory support, with a lower inotrope score, and without myocardial infarction as the cause of cardiogenic shock. These patients were successfully stabilized with low in-hospital mortality. Impella support, on the other hand, was chosen for patients in cardiogenic shock who were less stable, had a higher inotrope score, required greater ventilatory support, and had worse renal function. Patients in the Impella group had a longer median duration of support but comparable complications to patients on IABP support. While patients on Impella support had higher in-hospital mortality, despite the worse cardiogenic shock in patients for whom clinicians chose Impella support, over 75% were successfully stabilized to recovery or transplantation.

Randomized controlled trials of IABP vs. Impella support in cardiogenic shock have resulted in statistically similar clinical outcomes between the two devices [1,2,3] but it is not clear whether these findings are generalizable to all patients with cardiogenic shock due to several limitations. First, these trials comprised a limited number of patients, 26 [1] and 48 [2,3] patients, respectively. Second, these trials enrolled only patients with post-myocardial infarction cardiogenic shock and utilized the Impella 2.5 [1] and Impella CP [2,3] which offer inferior circulatory support compared to the contemporary Impella 5.5. With the noted caveats, while one trial found that patients randomized to Impella support had a higher cardiac index after 30 min compared to those randomized to IABP, 30-day mortality was no different [1]. Similarly, the other randomized trial observed comparable mortality between patients randomized to Impella and IABP at 30 days, 6 months [2], and 5 years [3].

On the other hand, a retrospective cohort study also focusing only on patients with post-myocardial infarction cardiogenic shock noted that patients with Impella support had higher rates of major bleeding and in-hospital mortality [4], which may reflect confounding by indication: patients who receive Impella versus IABP support at the clinician’s discretion are more unstable.

When assessing the impact of Impella versus IABP support in all patients with cardiogenic shock awaiting HTx, not restricted to just those with post-myocardial infarction, only observational data are available. In an analysis of the United Network of Organ Sharing (UNOS) registry, Impella versus IABP support did not impact post-transplant survival. However, patients who received Impella support were sicker with higher use of preoperative ventilation and higher risk of waitlist delisting compared with IABP-supported candidates, though there were no differences in post-transplant survival [5]. While randomized controlled trials are the gold standard for the assessment of causation, and large registry analyses offer the benefit of a large sample size with the power to detect differences and widespread applicability, a single-center analysis offers the distinct advantage of a detailed and granular assessment. In this case, we can provide a unique perspective: (1) highlighting the role of the clinician’s choice in selecting contemporary Impella 5.5 for patients with worse cardiogenic shock; and (2) describing the overall trajectory of patients with advanced heart disease who require tMCS, rather than focusing only on those who are waitlisted for HTx, as in the UNOS registry analysis, thereby reducing survival bias.

The triage of patients with cardiogenic shock to Impella versus IABP support is a complex decision that is based on the patient’s stability and clinical course. The improved outcomes in patients with IABP support were undoubtedly related to their greater relative stability at the time of tMCS implantation. The importance of clinician discretion in the triage of patients with cardiogenic shock to tMCS indicates the difficulty of factoring heterogeneity of patient presentations in randomized controlled trials of tMCS devices. Based on this study design, the criteria by which clinicians chose Impella vs. IABP remains unclear and based on individualized judgment and experience rather than a set clinical algorithm.

The limitations of our study include a single-center design in which patients were cared for by a select cohort of clinicians, which may limit generalizability. The patient population was highly selected to be cared for by advanced heart failure specialists and comprised very few patients with acute myocardial infarction as the cause of cardiogenic shock and ventilatory support prior to tMCS placement. Another limitation is that without randomization, the results are subject to confounding by indication as those factors that led clinicians to choose Impella support for their patients may have also influenced outcomes. However, the purpose of this study was not to determine the impact of Impella vs. IABP on outcomes but to provide an evaluation of clinician-guided therapy to survey the practices of real-world clinicians as they care for patients with cardiogenic shock and inform future shared decision making as patients are stabilized and the candidacy for advanced heart failure therapies is assessed.

## 5. Conclusions

In patients with cardiogenic shock at a high-volume advanced heart failure center, patients for whom their clinicians chose Impella versus IABP support for cardiogenic shock were more unstable at the time of tMCS implantation. Nonetheless, the majority were successfully stabilized and either recovered or survived to heart transplantation, indicating that clinicians perform important triage decisions on an individualized basis. This study offers preliminary insight from a highly selected sample into the real-world clinical characteristics, complications, and outcomes to guide clinicians in this complex decision-making process.

## Figures and Tables

**Table 1:** Clinical characteristics.

	IABP Group (*n* = 59)	Impella Group (*n* = 31)	*p*-Value
Age (years)	60 (51.5–67)	56 (48–64.5)	0.140
Female	14 (23.7%)	2 (6.5%)	0.047
Height (cm)	173 (165–180)	178 (174–183)	0.022
Weight (kg)	71 (61–88)	94 (79–105)	<0.001
BMI (kg/m^2^)	24.2 (22.1–27.4)	30.2 (24.8–32.6)	<0.001
Blood Type			
A	21 (35.6%)	7 (22.6%)	0.239
B	10 (17.0%)	9 (29.0%)	0.276
AB	1 (1.7%)	2 (6.5%)	0.272
O	27 (45.8%)	13 (42.0%)	0.825
Race/Ethnicity			
Caucasian	25 (42.4%)	12 (38.7%)	0.823
African American	8 (13.6%)	7 (22.6%)	0.373
Hispanic	12 (20.3%)	2 (6.5%)	0.126
Asian	7 (11.9%)	3 (9.7%)	1.000
Other	7 (11.9%)	7 (22.6%)	0.225
Diabetes Mellitus	31 (52.5%)	14 (45.2%)	0.658
Type of Cardiomyopathy			
Nonischemic dilated	35 (59.3%)	22 (71.0%)	0.359
Ischemic	22 (37.3%)	9 (29.0%)	0.490
Restrictive/infiltrative	0	0	1.000
Congenital	1 (1.7%)	0	1.000
Other	1 (1.7%)	0	1.000
Prior transplant evaluation	6 (10.2%)	4 (12.9%)	0.732
Prior transplant listing	3 (5.1%)	1 (3.2%)	1.000
Primary etiology of cardiogenic shock			
Acute decompensated HF	58 (98.3%)	26 (83.9%)	0.017
Acute myocardial infarction	1 (1.7%)	5 (16.1%)	0.017
Postcardiotomy shock	0	0	1.000
Myocarditis	0	0	1.000
Transplant rejection	0	0	1.000
Systolic blood pressure, mm Hg	88 (83–95)	91 (83–101)	0.368
Hemodynamics			
RA, mm Hg	13 (9–19)	17 (15–19)	0.360
PA mean, mm Hg	34 (28–40)	35 (28–39)	0.885
PCWP, mm Hg	23 (17–27)	25 (20–29)	0.297
CI, L/min/m^2^	1.9 (1.6–2.5)	2.1 (1.8–2.5)	0.188
INTERMACS Profile			
Profile 1	4 (6.8%)	3 (9.7%)	0.688
Profile 2	33 (55.9%)	21 (67.7%)	0.366
Profile 3	22 (37.3%)	7 (22.6%)	0.235
Inotrope use			
Inotrope score *	6.0 (2.0–7.6)	6.9 (5.9–10.8)	0.005
Dobutamine, mcg/kg/min	3 (3–4.9)	3 (2.8–5.0)	0.243
Milrinone, mcg/kg/min	0.25 (0.25–0.38)	0.25 (0.25–0.25)	0.186
Epinephrine, mcg/min	10 (8–12.5)	5 (2.8–7)	0.221
Dopamine, mcg/kg/min	3 (3–3.5)	5 (3.5–6)	0.155
Norepinephrine, mcg/min	9 (5.5–24.5)	7 (6–7)	0.089
Cardiopulmonary resuscitation	1 (1.7%)	0	1.000
Ventilator support	0	5 (16.1%)	0.004
Continuous renal replacement therapy	2 (3.4%)	3 (9.7%)	0.335
Ejection fraction (%)	15 (12–19)	16 (10–20)	0.765
Creatinine if not on dialysis (mg/dL)	1.2 (0.9–1.6)	1.9 (1.2–2.7)	0.005
Total bilirubin (mg/dL)	1.4 (0.8–1.9)	1.7 (1.2–2.9)	0.173
Axillary access (vs. femoral)	4 (6.8%)	31 (100%)	<0.001
Lactate (mmol/L)	1.2 (0.9–1.7)	1.2 (0.9–1.7)	0.795
Surgical cut-down (vs. percutaneous)	4 (6.8%)	31 (100%)	<0.001

* Inotrope score was compared only for those on inotropes. The inotrope score was calculated as follows: dopamine (×1) + dobutamine (×1) + amrinone (×1) + milrinone (×15) + epinephrine (×100) + norepinephrine (×100) with each drug dosed in μg/kg/min. BMI, body mass index; CI, cardiac index; PA, pulmonary artery; PCWP, pulmonary capillary wedge pressure; RA, right atrial.

**Table 2:** Temporary MCS complications.

All Events Per 100 Person-Years	IABP Group (*n* = 59)	Impella Group (*n* = 31)	*p*-Value
Bleeding adverse events	582.4	1568.6	0.035
Limb ischemia	79.4	0.0	<0.001
Pseudoaneurysm	0.0	0.0	1.000
AV * fistula	0.0	0.0	1.000
Vessel thrombosis/distal embolization	0.0	0.0	1.000
Vessel dissection, perforation, or rupture	0.0	0.0	1.000
Vessel stenosis	0.0	0.0	1.000
Cannulation site bleeding	493.6	1404.8	0.060
Vascular access site infection	0.0	0.0	1.000
Major hemolysis	80.3	1776.3	<0.001
Minor hemolysis	250.7	772.2	0.058
Infection adverse event	797.9	1400.7	0.173
Stage 3 AKI	162.7	756.4	0.008
Need for RRT due to stage 3 AKI	159.5	617.6	0.028
Need for RRT due to chronic renal dysfunction	281.0	60.8	<0.001
Type 1 neurologic dysfunction adverse event (acutely symptomatic CNS injury)	0.0	0.0	1.000
Major device malfunction	161.3	327.3	0.189

* AV, arteriovenous; AKI, acute kidney injury; CNS, central nervous system; RRT, renal replacement therapy.

**Table 3:** Outcomes.

	IABP Group (*n* = 59)	Impella Group (*n* = 31)	*p*-Value
Duration of support (d)	7 (4–10)	15 (10–26.5)	<0.001
Recovery	10 (17.0%)	3 (9.7%)	0.530
Durable MCS	2 (3.4%)	1 (3.2%)	1.000
HeartWare LVAD	0	0	1.000
HeartMate 3 LVAD	2 (3.4%)	1 (3.2%)	1.000
Total Artificial Heart	0	0	1.000
Heart transplantation	45 (76.3%)	21 (67.7%)	0.455
Death during admission	2 (3.4%)	6 (19.4%)	0.018
Death	6 (10.2%)	8 (25.8%)	0.068

## Data Availability

Data supporting reported results can be provided upon reasonable request.
